# Molecular genetic analysis of *PKHD1* by next-generation sequencing in Czech families with autosomal recessive polycystic kidney disease

**DOI:** 10.1186/s12881-015-0261-3

**Published:** 2015-12-22

**Authors:** Lena Obeidova, Tomas Seeman, Veronika Elisakova, Jana Reiterova, Alena Puchmajerova, Jitka Stekrova

**Affiliations:** Institute of Biology and Medical Genetics of the First Faculty of Medicine, General University Hospital in Prague, Prague, Czech; Department of Paediatrics, 2nd Faculty of Medicine, Charles University in Prague and Motol University Hospital, Prague, Czech; Department of Nephrology, First Faculty of Medicine, Charles University in Prague and General University Hospital in Prague, Prague, Czech; Department of Biology and Medical Genetics, 2nd Faculty of Medicine, Charles University in Prague and Motol University Hospital, Prague, Czech

**Keywords:** Autosomal recessive polycystic kidney disease, ARPKD, PKHD1, Mutation analysis, Next-generation sequencing

## Abstract

**Background:**

Autosomal recessive polycystic kidney disease (ARPKD) is an early-onset form of polycystic kidney disease that often leads to devastating outcomes for patients. ARPKD is caused by mutations in the *PKHD1* gene, an extensive gene that encodes for the ciliary protein fibrocystin/polyductin. Next-generation sequencing is presently the best option for molecular diagnosis of ARPKD. Our aim was to set up the first study of ARPKD patients from the Czech Republic, to determine the composition of their mutations and genotype-phenotype correlations, along with establishment of next-generation sequencing of the *PKHD1* gene that could be used for the diagnosis of ARPKD patients.

**Methods:**

Mutational analysis of the *PKHD1* gene was performed in 24 families using the amplicon-based next-generation sequencing (NGS) technique. In patients without 2 causal mutations identified by NGS, subsequent MLPA analysis of the *PKHD1* gene was carried out.

**Results:**

Two underlying mutations were detected in 54 % of families (*n* = 13), one mutation in 13 % of families (*n* = 3), and in 33 % of families (*n* = 8) no mutation could be detected. Overall, seventeen different mutations (5 novel) were detected, including deletion of one exon. The detection rate in our study reached 60 % in the entire cohort of patients; but 90 % in the group of patients who fulfilled all clinical criteria of ARPKD, and 42 % in the group of patients with unknown kidney pathology. The most frequent mutation was T36M, accounting for nearly 21 % of all identified mutations.

**Conclusions:**

Next-generation sequencing of the *PKHD1* gene is a very useful method of molecular diagnosis in patients with a full clinical picture of ARPKD, and it has a high detection rate. Furthermore, its relatively low costs and rapidity allow the molecular genetic analysis of patients without the full clinical criteria of ARPKD, who might also have mutations in the *PKHD1* gene.

## Background

Autosomal recessive polycystic kidney disease (ARPKD, OMIM #263200) is a severe form of polycystic kidney disease, with an estimated incidence of 1:20,000 [1]. Its histological features are comprised of fusiform dilatations of the renal collecting ducts, and ductal plate malformation causing biliary ectasia and hepatic fibrosis. The clinical presentation of ARPKD is highly variable, and even thought the majority of cases are identified *in utero* or at birth, cases of adult and elderly patients with ARPKD have also been described [2–4]. The most severely affected fetuses display enlarged echogenic kidneys and oligohydramnios, caused by decreased fetal urine output that can result in a critical degree of pulmonary hypoplasia. Arterial hypertension affects up to 80 % of children with ARPKD and usually develops within the first months of life [4, 5]. The early phenotype of ARPKD is primarily affected by renal defects and their co-morbidities, while liver impairment with consecutive portal hypertension dominates the phenotype in older patients.

ARPKD is caused by mutations in the *PKHD1* gene, located on chromosome 6p12 and extending over 470 kb of the genomic sequence [6, 7]. Its longest reading frame (ORF) comprises 66 exons, and encodes a large (4074 amino acids) single-pass transmembrane protein fibrocystin/polyductin. Although the exact function of fibrocystin in the formation of the ARPKD phenotype remains unclear, localization of fibrocystin in primary cilia and its interaction with cation channel polycystin-2 suggests its participation in signaling pathways, disruption of which can cause defects in morphogenesis and maintenance of tubular architecture in the organs [8–10].

The severity of the ARPKD phenotype and its formation in the perinatal period result in a strong demand for reliable prenatal diagnosis. Although second-trimester sonography is usually sufficient to suggest the diagnosis of ARPKD, typical findings such as enlarged echogenic kidneys and oligohydramnios may not yet be visible [11]. Molecular genetic analysis is therefore a possible choice for early (and the only reliable) prenatal diagnosis. Thanks to the advance in techniques of molecular analysis, next-generation sequencing (NGS) is now the state-of-the-art method replacing haplotype-based linkage analysis and Sanger sequencing, with or without mutation pre-screening.

Nevertheless, even with the advantages of NGS-based approaches, the molecular diagnostics of ARPKD still poses numerous challenges. *PKHD1* is a large gene with high allelic heterogeneity and a great number of unique mutations. To date, 748 variants of the *PKHD1* gene have been described in ARPKD/PKHD1 Aachen database [http://www.humgen.rwth-aachen.de/]. Such a high number of new variants cause problems with genotype-phenotype correlation, and predictions of the pathogenicity of novel missense mutations must be carried out with caution. Moreover, mutations are spread all over the *PKHD1* gene, without mutational hotspots, which makes molecular analysis relatively time-consuming and costly.

Molecular diagnosis of ARPKD is also complicated by its relatively broad range of clinical manifestations that can be mimicked by mutations in other (ciliary) genes. Polycystic kidney disease (PKD) with an early and severe phenotype can be seen in about 2 % of patients with typical late-onset autosomal dominant PKD [12]. These patients can be phenotypically indistinguishable from ARPKD patients, but they harbor multiple mutations in ADPKD genes *PKD1* and/or *PKD2*; in rare cases in combination with mutations in another gene, *HNF1β/TCF2* (hepatocyte nuclear factor 1β/transcription factor 2) [12].

Our study was performed within the research project in order to provide a first glimpse of the composition of mutations in Czech ARPKD patients (no study on this topic had previously been done in the Czech Republic), to determine the phenotype-genotype correlations in patients together with gradually setting up molecular analysis of the *PKHD1* gene by amplicon-based next-generation sequencing.

Herein, we present molecular genetic analysis of the *PKHD1* gene by amplicon-based next-generation sequencing and subsequent MLPA (Multiplex Ligation-dependent Probe Amplification) in 24 families from the Czech Republic.

## Methods

### Patients and samples

Mutational analysis of the *PKHD1* gene was carried out in 24 Czech families, and the set analyzed by NGS counted 26 samples. The cohort of probands was divided into two groups (A and B) on the basis of their fulfillment of established clinical criteria of ARPKD including: 1) typical kidney involvement on ultrasound (US) (enlarged hyperechogenic kidneys with bilateral poor corticomedullary differentiation); 2) typical liver involvement (congenital hepatic fibrosis, ductal plate malformation); and 3) normal renal US of both parents, consistent with autosomal-recessive inheritance [13, 14]. Patients fulfilling all three criteria were placed in Group A, which consisted of 10 patients: 9 children (mean age 8.3 ± 4.9) and 1 adult patient who was clinically diagnosed in his childhood and now plans a family. Group B consisted of 16 samples coming from 14 families: 7 children (2 of them were brothers), mean age 6.2 ± 4.9, with cystic kidney disease of unknown etiology; 3 severely affected newborns with bilateral massively enlarged kidneys with a number of small cysts who died shortly after the birth; 4 fetuses from terminated pregnancies (DNA of one fetus was not available, therefore the NGS analysis was carried out for both parents of this fetus), with oligo/anhydramnios and renal pathology including dilated tubules and/or collecting ducts, changed kidney size (in 3 cases enlarged kidneys, in 1 case hypoplastic kidneys) found prenatally - in 2 cases with the presence of multiple small cysts, and 1 sample of a fetus obtained prenatally by amniocentesis with normohydramnios but bilaterally enlarged hyperechogenic kidneys. Detailed clinical information about the patients is summarized in Tables 1 and 2. The samples of probands and their parents were provided by several hospitals across the Czech Republic. The study was approved by the Ethics Committee of the General University Hospital in Prague, and all patients/legal guardians were consulted by a geneticist prior to molecular-genetic analysis, with all having given written informed consent for the genetic testing.

**Table 1:** Summary of mutations and clinical phenotype of patients within Grup A

Patient/Family	Mutation	M/P	Age at diagnosis	Parental renal ultrasound	Renal ultrasound	Hepatic pathology (e.g. CHF) (Yes/No)^a^	HTN^b^	eGFR^c^ (ml/min/1.73 m^2^)
1567	R328Q G2705VfsX11 S2861G	P M M	Perinatal	Normal	Hyperechogenic enlarged kidneys with poor cortico-medullary differentiation	Yes (Caroli’s syndrome)	Yes (drugs)	N/A (after renal transplantation)
893	L1966TfsX4 G2705VfsX11 S2861G	P M M	Infantile (6 months)	Normal	Hyperechogenic enlarged kidneys with poor cortico-medullary differentiation	Yes (US)	Yes (drugs)	72
1358	T36M A2521FfsX59	M -	Perinatal	Normal	Hyperechogenic enlarged kidneys with poor cortico-medullary differentiation	Yes (US and MRI)	Yes (drugs)	22
1177	T36M I3553T	M P	Perinatal	Normal	Hyperechogenic enlarged kidneys with poor cortico-medullary differentiation	Yes - hyperechogenic structure (US)	Yes (drugs)	53
1359	R1624W R1775X	P M	Perinatal	Normal	Hyperechogenic enlarged kidneys with poor cortico-medullary differentiation	Yes (US)	Yes (drugs)	91
124	R1624W L1966TfsX4	Nonpaternity M	Infantile (3 years)	Normal	Hyperechogenic enlarged kidneys, small cysts in cortex, calicolithiasis suspicious, postnatal micro- and macrocysts	Yes (US)	Yes (ABPM, drugs since 3 y)	118
1085	L1966TfsX4 G2705VfsX11 S2861G	P M M	Perinatal	Normal	Enlarged kidneys with multiple cysts	Yes - hyperechogenic structure (US)	Yes (drugs)	52
1006	W937X R1624W	M -	Childhood (8 years)	Normal	Hyperechogenic enlarged kidneys with poor cortico-medullary differentiation	Yes - periportal fibrosis (US)	Yes (drugs)	N/A (after renal transplantation)
1479	G2705VfsX11 S2861G	P P	Infantile (15 months)	Normal	Hyperechogenic enlarged kidneys with poor cortico-medullary differentiation	Yes (US and also biopsy)	Yes (drugs)	76
605	T36M	M	Infantile (2 months)	Normal	Hyperechogenic enlarged kidneys with poor cortico-medullary differentiation	Yes (US)	Yes (drugs)	71

*ABPM* Ambulatory blood pressure monitoring, *CHF* Congenital hepatic fibrosis, *CKD* Cystic kidney disease, *eGFR* Estimated glomerular filtration rate, *ESRD* End-stage renal disease, *HTN* Hypertension, *M* Maternal, *MRI* Magnetic resonance imaging, *N/A* Not applicable, *P* Paternal, *S-creatinine* Serum creatinine, *US* Ultrasound, ^a^CHF was diagnosed by US and/or MRI, or on the basis of biopsy or autopsy, ^b^Hypertension was defined as usage of antihypertensive drugs or/and blood pressure equal or above 95th percentile, ^c^eGFR according to Schwartz (ml/min/1.73 m^2^)

**Table 2:** Summary of mutations and clinical phenotype of patients within Grup B

Patient/Family	Mutation	M/P	Age at diagnosis/Death	Parental renal US	Renal pathology	Hepatic pathology	Prenatal findings	Additional postnatal/autopsy findings	HTN^a^	eGFR^b^ (ml/min/1.73 m^2^)
1388	T36M G770DfsX6	P M	Prenatal/perinatal death	Normal	Enlarged kidneys with multiple microcysts	Yes	Oligohydramnios, infantile polycystic kidney disease	Pneumothorax on the right site, hypoxic-ischemic encephalopathy, mild club foot	N/A	N/A
1513	T36M R2671X	M de novo	Perinatal/perinatal death	Normal	Enlarged kidneys with multiple microcysts, two renal arteries on both sides	Yes	Enlarged kidneys	Bilateral pneumothorax, pulmonary hypoplasia - respiratory insufficiency	N/A	N/A
1052	T36M R124X	M P	Prenatal (TOP)	Normal	Enlarged kidneys with multiple microcysts	No	Anhydramnios, infantile polycystic kidney disease	--	N/A	N/A
600	H2931P Exon deletion	M P	Prenatal/perinatal death	Normal	Enlarged kidneys with multiple microcysts	Yes (CHF)	Anhydramnios	Potter sequence, cerebral edema	N/A	N/A
446	Q905X	M	Prenatal (TOP)	Not available	Enlarged kidneys with dilated tubules in cortex and medulla	No	Oligohydramnios, infantile polycystic kidney disease	Mild pulmonary hypoplasia	N/A	N/A
974	No	N/A	Prenatal (TOP)	Normal	Hypoplastic kidneys with cystically dilated collecting ducts	No	Anhydramnios	Club foot, Potter facies, probable umbilical cord thrombosis	N/A	N/A
1629	No	N/A	Prenatal (TOP)	Mother, maternal grandmother/aunt/cousin - polycystic kidneys	Bilaterally massively enlarged kidneys with multiple microcysts	No	Oligohydramnios, bilaterally massively enlarged kidneys, pulmonary hypoplasia	--	N/A	N/A
883	No	N/A	Prenatal	Mother - normal	Bilaterally enlarged hyperechogenic kidneys	--	Normohydramnios, bilaterally enlarged hyperechogenic kidneys	--	N/A	N/A
460, 461	I222V P755L	P M	460: childhood (6 years) 461: prenatal	Normal	460: slightly enlarged kidneys, hyperechogenic cortex with areas with microcysts 461: bilaterally enlarged hyperechogenic kidneys with micro and macrocysts	460: No 461: No	460: N/A 461: oligohydramnios, prenatal US: normal	--	460: No 461: No	460: 151 461: 90
1182	No	N/A	Perinatal	Normal	Hyperechogenic normal size kidneys	Yes (US)	N/A	--	Yes (drugs)	79
1178	No	N/A	Perinatal	Normal	Hyperechogenic enlarged kidneys with poor cortico-medullary differentiation and macrocysts	No	N/A	--	Yes (drugs)	N/A (after renal transplantation)
889	No	N/A	Prenatal	Normal	Polycystic kidneys on prenatal US	No	Polycystic kidneys	--	Yes (borderline)	not available
1340	No	N/A	Perinatal	Normal	Enlarged kidneys with multiple nodulary hyperplastic structures (susp. nephroblastomatosis)	No (MRI)	Polyhydramnios	--	Yes (drugs)	111
1371	No	N/A	Infantile (6 months)	Mother and maternal grandmother ADPKD	Multiple macrocysts in both normal size kidneys	No (but acquired micronodular liver cirrhosis on biopsy)	N/A	--	No	142

*CHF* Congenital hepatic fibrosis, *M* Maternal, *N/A* Not applicable, *P* Paternal, *TOP* Termination of pregnancy, *US* Ultrasound, ^a^Hypertension was defined as usage of antihypertensive drugs or/and blood pressure equal or above 95th percentile,^b^eGFR according to Schwartz (ml/min/1.73 m^2^)

### *PKHD1* mutation analysis

The *PKHD1* library for next-generation sequencing counted 87 amplicons, and was prepared in two steps with a universal-tailed amplicon sequencing design. Target-specific universal-tailed fusion primers (Generi Biotech) used for the first round of PCR have been described previously in the literature [15]. The library was purified by Agentcourt® AMPure® XP (Beckman Coulter), and the final library of 6 patients was mixed regarding their concentration (Quant-iT™ PicoGreen® dsDNA Assay Kit, Invitrogen), and then prepared for a sequencing run on a GS Junior with a GS Junior Titanium emPCR Kit (Lib-A) and a GS Junior Titanium Sequencing Kit (Roche Diagnostics). The data were analyzed using the Sequence Pilot program (JSI Medical Systems GmbH). All probable causal mutations found by NGS were confirmed in patients by Sanger sequencing done on an ABI PRISM 3130 Genetic Analyzer (Life Technologies); targeted Sanger sequencing was also performed in the parents/relatives of patients to determine the position of mutations at homologous chromosomes. Subsequent MLPA analysis (Multiplex ligation-dependent probe amplification) was done for all patients without 2 causal mutations found by NGS. The kits used for MLPA analysis were SALSA MLPA P341 PKHD1 mix 1, and P342-PKHD1 mix 2 (MRC Holland). The software used for the analysis of MLPA data was Coffalyser.Net (MRC Holland).

### Data analysis and sequence changes classification

All sequenced samples were compared to the reference sequence of the *PKHD1* gene [NM_138694.3]. Changes found within this study were checked against the Mutation Database Autosomal Recessive Polycystic Kidney Disease (http://www.humgen.rwth-aachen.de) and/or the Human Gene Mutation Database (http://www.hgmd.cf.ac.uk/ac/index.php). In the case of novel mutations, their pathogenic potential was assessed. Nonsense or frameshift variants leading to a premature STOP codon, as well as exon deletions, were considered as definitely pathogenic. The pathogenic potential of new missense variants was evaluated computationally using PolyPhen-2 [16] and MutationTaster [17] . Two other programs, NetGene2 [18, 19] and Human Splicing Finder [20], were used to assess any possible splice effect of intronic variants. Common polymorphisms were checked against the Aachen PKHD1 database (see above) and/or the NCBI SNP database (http://www.ncbi.nlm.nih.gov/projects/SNP/).

## Results

We performed mutational analysis of the longest ORF of the *PKHD1* gene in a set of 26 samples from 24 unrelated families. The analysis consisted of amplicon-based NGS, with subsequent MLPA analysis in those cases where less then two mutations were found on NGS. In Group A (clinically diagnosed ARPKD), two underlying mutations were detected in 8 out of 10 families (80 %), and one mutation in 2 families (20 %). Thus, the overall detection rate amounts to 90 % in Group A. In Group B (cystic kidney diseases of unknown etiology), two underlying mutations were detected in 5 out of 14 families (36 %), one mutation in 1 family (7 %), and no mutations could be detected in 8 families (57 %). Thus, the overall detection rate is 42 % in Group B. The overall detection rate for the entire patient cohort is 60 %.

Regarding the types of mutations found in the entire cohort, 14 (48 %) were truncating mutations (formed by nonsense (*n* = 5) and frameshift (*n* = 9) changes), and 14 (48 %) were missense changes. Additionally, one new exon deletion has been found (Table 3).

**Table 3:** All causal mutations found in our cohort of patients

Exon	cDNA level	Protein level	Allele frequency % (*n* = 52)	Reference
3	c.107C > T	p.Thr36Met	11.5	[7]
5	c.370C > T	p.Arg124X	1.9	[24]
9	c.664A > G	p.Ile222Val	3.8	[7]
14	c.983G > A	p.Arg328Gln	1.9	[25]
22	c.2264C > T	p.Pro755Leu	3.8	[4]
**23**	**c.2309delG**	**p.Gly770AspfsX6**	**1.9**	**Novel**
**25**	**c.2713C > T**	**p.Gln905X**	**1.9**	**Novel**
26	c.2810G > A	p.Trp937X	1.9	[22]
32	c.4870C > T	p.Arg1624Trp	5.8	[6]
33	c.5323C > T	p.Arg1775X	1.9	[4]
36	c.5895dupA	p.Leu1966ThrfsX4	5.8	[7]
**48**	**c.7561_7568del8**	**p.Ala2521PhefsX59**	**1.9**	**Novel**
50	c.8011C > T	p.Arg2671X	1.9	[7]
51	c.8114delG	p.Gly2705ValfsX11	7.7	[24]
**56**	**c.8792A > C**	**p.His2931Pro**	**1.9**	**Novel**
61	c.10658 T > C	p.Ile3553Thr	1.9	[7]
**62**	**Deletion of the exon**		**1.9**	**Novel**

New variants are showed in bold. Allele frequency was counted for current study

**Fig. 1:**
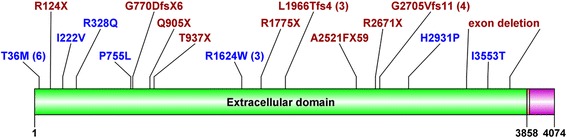
Schematic representation of fibrocystin with indicated localization of mutations detected in our study. Protein domains: green – extracellular domain, pink – transmembrane domain, violet – intracellular domain. The number of occurrence is shown for recurrent mutations. Missense mutations are shown in blue, truncating mutations in red [33]

In total, 17 different mutant variants, almost evenly distributed throughout the predicted extracellular domain of fibrocystin, were found within this study (Fig. 1). Five changes were novel (including an exon deletion), and twelve changes had already been described in either one of the mutational databases mentioned above. Three of the novel mutations were considered as pathogenic because they cause formation of the premature stop codon. Also, the whole exonic deletion was considered as potentially causal. Missense change p.His2931Pro was run through by two prediction programs (PolyPhen-2 and MutationTaster), with both predicting its probable pathogenicity.

The majority of mutant alleles were unique for each family (*n* = 13, I222V, and P755L were also considered as unique as they were present in brothers); however, four variants (p.Thr36Met, p.Arg1624Trp, p.Leu1966ThrfsX4, and p.Gly2705ValfsX11 - within exons 3, 32, 36, and 51, respectively) appeared in several families and formed a substantial part (55 %) of all mutations found in this study. The most frequent change was p.Thr36Met, located in exon 3; having been detected in 6 families, and thus accounting for almost 21 % of all mutations found.

All patients with 2 causal mutations of the *PKHD1* gene were compound heterozygotes; except for two patients who seemed to have one mutation which had arisen *de novo* (Tables 1, 2). Nevertheless, this was only confirmed in the first patient (patient 1513), since non-paternity was confirmed in the second patient (patient 124). We can speculate the *de novo* mutation in patient 1513 was inherited paternally, since the father of the affected child was 54 years old when the child was born, and it is well known that the number of new mutations passed on to a child rises exponentially with paternal age [21]. Still, the germline mosaicism (in both of the parents) could possibly be the cause of the mutation in this child. The origin of one mutation in two patients (1358, 1006) could not be assessed as only the maternal DNA was available.

The majority of patients with two causal mutations harbored a combination of a missense and chain-terminating mutation (*n* = 8); while the combination of two missense or two truncating mutations was accounted for by two patients each. In one patient, a combination of a missense mutation and an exon deletion was described.

The same combination of two changes in cis was detected in four our patients (1567, 893, 1085, 1479). One of the changes was known mutation p.Gly2705ValfsX11, while the second one was p.Ser2861Gly (rs150925674). The latter had appeared a few times in the literature, sometimes with the pathogenic mutations located on the same allele [4, 22–24]. The same combination as ours was even described in two patients in an article by Gunay-Aygun et al. [23]. Three of these families were consulted regarding their possible kinship, with no relationships discovered. Moreover, we performed haplotype analysis using microsatellite markers (D6S1662, D6S272, D6S1714, and D6S436) within these three families, and again no kinship was demonstrated. Therefore, it is possible these two changes are in linkage. As p.Ser2861Gly often appears together with a pathogenic mutation in cis, and also with a causal mutation at the second allele, it is considered as most probably non-pathogenic, which is supported by both prediction programs used in this study, thus determining the change as a benign polymorphism.

In addition to the causal mutations, we also detected a number of single nucleotide polymorphisms, whether causing amino acid substitution or silent changes (Table 4). Moreover, changes in intronic sequences - previously described or novel - were found (Table 5). Nevertheless, all of these new intronic changes were quite far from the exon-intron boundaries to disrupt the splite site, and both used prediction programs predicted their putative non-pathogenity.

**Table 4:** All exonic polymorphisms found in our study

Exon	cDNA level	Protein level	rs number	Allele frequency % (*n* = 52)
4	c.214C > T	p.Leu72Leu	rs6901799	13.5
4	c.234C > T	p.Asp78Asp	rs9474143	32.7
15	c.1185 T > C	p.Asp395Asp	rs1896976	100.0
17	c.1587 T > C	p.Asn529Asn	rs62406036	9.6
19	c.1736C > T	p.Thr579Met	rs45500692	1.9
**20**	**c.1929C > A**	**p.Thr643Thr**		**1.9**
20	c.1950G > A	p.Arg650Arg	rs143226202	1.9
21	c.2046A > C	p.Pro682Pro	rs4715271	13.5
22	c.2278C > T	p.Arg760Cys	rs9370096	38.5
24	c.2489A > G	p.Asn830Ser	rs62406032	9.6
32	c.3756G > C	p.Leu1252Leu	rs9689306	7.7
32	c.3785C > T	p.Ala1262Val	rs9296669	38.5
35	c.5608 T > G	p.Leu1870Val	rs2435322	100.0
48	c.7587G > A	p.Gly2529Gly	rs12210295	63.5
48	c.7673G > A	p.Arg2558Gln	rs369677008	1.9
49	c.7764A > G	p.Leu2588Leu	rs9349603	30.8
55	c.8581A > G	p.Ser2861Gly	rs150925674	7.7
58	c.9237G > A	p.Ala3079Ala	rs765525	46.2
61	c.10521C > T	p.His3507His	rs34460237	9.6
63	c.11340 T > C	p.Pro3780Pro	rs17667728	3.8
66	c.11696A > G	p.Gln3899Arg	rs4715227	48.1
66	c.11714 T > A	p.Ile3905Asn	rs2661488	3.8
67	c.11878G > A	p.Val3960Ile	rs34548196	3.8
67	c.12143A > G	p.Gln4048Arg	rs9381994	25.0

New polymorphism is in bold

**Table 5:** All intronic variants (IVS) found in our group of patients

Intron	cDNA level	rs number	Allele frequency % (*n* = 52)
7	c.527 + 19 T > C	rs17577001	32.7
7	c.527 + 51G > T	rs62406055	9.6
**8**	**c.528-99delG**		**11.5**
8	c.602 + 67A > G	rs3936986	38.5
**12**	**c.779-30C > T**		**9.6**
16	c.1234-10 T > A	rs4715272	17.3
19	c.1694-32C > G		5.8
20	c.1964 + 17G > T	rs201349527	1.9
23	c.2407 + 50C > T	rs10948667	38.5
31	c.3561-22A > G		1.9
32	c.3629-32A > G	rs2499480	44.2
32	c.5236 + 14A > G	rs12210725	5.8
49	c.7734-4 T > C	rs7452724	30.8
52	c.8302 + 12 T > A	rs1571084	48.1
52	c.8302 + 18A > G	rs12529717	1.9
**53**	**c.8303-65C > A**		**71.2**
53	c.8440 + 71A > G	rs80047024	5.8
54	c.8441-32G > C	rs3920621	46.2
56	c.8643-72C > T	rs9370049	36.5
57	c.8798-19A > C	rs1326605	75.0
61	c.11174 + 11A > G	rs115072237	3.8
67	c.11786-30C > T	rs9395699	15.4

Newly described IVS are in bold. Both prediction programs (see Methods) determined the new IVS have no impact on splice site abruption and are therefore probably without disease-causing significance

## Discussion

The classical molecular genetic analysis of the *PKHD1* gene, consisting of Sanger sequencing with/without previous usage of a mutation screening method, is rarely performed in routine diagnostics due to its comprehensiveness as well as being both quite time- and cost-consuming. Also the wide range of ARPKD phenotypes, from severely affected fetuses to adult patients with milder symptoms, makes the decision about a molecular analysis of a patient even harder. Nevertheless, successful genetic analysis can help in management of the disease and also in future family planning.

The aim of our study was to set up the molecular analysis of the *PKHD1* gene using the next-generation sequencing technique, and to perform a comprehensive study of *PKHD1* variants including their genotype-phenotype correlations in a group of Czech patients.

The analyzed group was composed of 24 families from all over the Czech Republic with suspected ARPKD. The detection rate for the whole cohort of families reached 60 %, and hence was slightly lower in comparison with published detection studies (reviewed by Gunay-Aygun et al. [23]). This was caused by the inclusion of patients with less strictly clinically defined and not perfectly fulfilling the “typical” features of ARPKD (*e.g.*, absence of congenital liver fibrosis, fetuses from terminated pregnancies etc. - Group B). In the group of patients who fulfilled all the diagnostic criteria for a clinical diagnosis of ARPKD, the detection rate was markedly higher, reaching 90 %. This detection rate is at least comparable with the detection rates from previously published studies from other countries [23].

However, the ARPKD phenotype has a wide spectrum of presentations. There were probands within our Group B who did not fulfill all diagnostic criteria for a clinical diagnosis of ARPKD phenotype; however, despite that, two causal mutations were detected. There is also the possibility that some of the patients without 2 causal mutations in *PKHD1* harbor a mutation located within its intronic region, with an impact on gene expression and/or mutations in other genes (*e.g.*, *PKD1*, *PKD2*, *HNF1β*, etc.) that could cause a severe renal and/or hepatic phenotype resembling ARPKD.

For this reason, we also included in our group of patients a girl from a family with clinically diagnosed autosomal dominant polycystic kidney disease (ADPKD), where linkage analysis of 8 family members (of which 7 were affected) showed that the disease was linked to the *PKD1* gene. Unlike the other affected members of the family, the girl (patient 1371) not only had numerous cysts in both kidneys, but also hepatomegaly with suspected congenital hepatic fibrosis. Hence, the possible concurrence of mutations in *PKD1*/*PKHD1* or *PKD1*/*HNF1β* genes was suggested. No mutation of the *PKHD1* gene was discovered in this girl, and the diagnosis of ARPKD was later ruled out when hepatic micronodular cirrhosis with steatosis was confirmed at liver biopsy, and congenital hepatic fibrosis was excluded. In conclusion, the girl has two diseases concurrently: ADPKD and hepatic cirrhosis.

Another family with ADPKD caused by mutation c.1340_1346dup7 in *PKD2* was also included in our studied group because the pregnancy of an affected mother with ADPKD was terminated, due to a prenatal finding showing bilateral massively enlarged kidneys of the fetus, caused by a large number of small cysts located in the Bowman’s capsule and tubules, as well as a severe form of pulmonary hypoplasia. Such a severe phenotype did not appear in the rest of the affected family members, and thus could indicate involvement of another gene mutation in the phenotype of a fetus. Nevertheless, no mutation of the *PKHD1* gene was detected in the fetus (patient 1629) and we would have to carry out mutation analysis of other ciliary genes to find the possible cause of such a severe phenotype of a fetus.

Our Group B included 7 patients with severe phenotypic manifestations (newborns that died shortly after birth and fetuses from termination of pregnancies - TOP) plus one sample of a fetus obtained by amniocentesis. Among these patients, 4 had two causal mutations found. Interestingly, 3 of these 4 patients harbored the combination of T36M and a truncating mutation. This combination also appeared in one patient (1358) with perinatally diagnosed ARPKD. This patient had congenital hepatic fibrosis, hypertension, and an advanced stage of cystic kidney disease at the age of nine. Our findings correspond to those made by both Bergmann et al. and Furu et al. [4, 22] that T36M in combination with some of the missense mutations or truncating mutation often cause a quite severe form of ARPKD. As described by Furu et al., T36M represents a potential alternative initiation codon predicted to be stronger than the native start site; causing formation of an abrupt protein without the sequence required for protein folding [22]. Therefore, such a mutation, despite being missense, can have an effect as a truncating mutation with complete loss of allele function. If so, T36M can be considered as a truncating mutation, and our results match the observations that practically all patients carrying 2 truncating mutations display a very severe phenotype, with nearly 100 % neonatal mortality. Additionally, the presence of at least 1 missense mutation is necessary for the patients to survive the neonatal period [22, 25, 26].

Three of the patients within this subgroup with severe manifestations had no mutation found. It is possible that these patients harbor a mutation in another ciliary gene, such as *HNF1β/TCF2, PKD1*, or *NPHP1-13*. Especially one fetus (sample 883) diagnosed at the 22nd week of gestation with findings showing hyperechogenic kidneys, but a normal amount of amniotic fluid, is a good candidate for mutational analysis of the *HNF1β/TCF2* gene as it was described in a paper by Decramer et al. [27], that *TCF2* gene mutations were detected in 29 % of 62 studied pregnancies with bilateral hyperechogenic kidneys of the fetus. Additionally, an antenatal phenotype of our fetus falls into two typical phenotypes described in Decramer’s study among patients with *TCF2* anomaly. The study by Decramer et al. also points out that patients with very early onset ADPKD (VEO-ADPKD) can also manifest with antenatal hyperechogenic kidneys; therefore, renal ultrasound of both parents to exclude VEO-ADPKD is essential for establishing a correct diagnosis in patients with antenatal hyperechogenic kidneys.

The mutations lie throughout the *PKHD1* gene, specifically over the part of the gene that encodes for the extracellular part of the protein (Fig. 1). Despite the high number of private mutations spread over the full length of the gene, few research groups have proposed an algorithm for an efficient molecular analysis to cut down expenses, or the time needed for a successful molecular genetic diagnosis in the daily routine [15, 28, 29]. Our group of patients was not so numerous so as to allow a potential suggestion of mutational analysis sequence among Czech patients. However, 4 recurrent mutations (T36M, R1624W, L1966TfsX4, and G2705VfsX11) formed a large portion (55 %) of all mutations found. Therefore, the analysis of only four exons of *PKHD1* containing these mutations (3, 32, 36, and 51, respectively) would achieve a detection rate of 33 %.

The T36M mutation within exon 3 was found 6 times within our group of patients, and thus comprised 21 % of all mutations found. Such a high prevalence of the T36M mutation is completely in accordance with the observation made by Bergmann et al. [28] that T36M constitutes approximately every fifth mutated *PKHD1* allele, and so occurs in every *PKHD1* mutational study [4, 13, 15, 22, 24, 25, 30, 31]. As for origin of this variant, it was suggested that it is either a founder allele (as most of the carriers are of European origin), or constitutes a mutational hot-spot. The allele sharing analyses made by Bergmann et al. showed that at least 18 different haplotypes are responsible for the prevalence of T36M in European populations, and that most likely T36M is located in a mutational hot-spot made up by mutagenic CpG dinucleotide [26]. The hypothesis that this allele occurs frequently, due to a mutational event, was favored in a study by Sharp et al. [24]. In this study, T36M was identified within diverse ethnic origins including African American and Egyptian; therefore, indicative of independent multiple mutational events. Nevertheless, as commented by Bergmann et al. [26], some alleles of T36M (especially in central Europe where T36M is quite frequent) might have a common ancestral origin. As the remainder of the mutations were all unique (they were detected in only one family), we would need a larger set of patients from the Czech Republic to potentially find additional common mutations among Czech patients with ARPKD that could be added to an exon panel. The results of our study clearly show how important a clinical evaluation of a patient’s phenotype is for an effective molecular analysis. One group of patients (A) who completely fulfilled the clinical criteria, including typical kidney and liver involvement as well as normal renal US of both parents (for details see Methods), almost always (8 families of 10) harbored 2 causal mutations in the *PKHD1* gene. In the remaining 2 families (patients 605 and 1479) one mutation was found, and there was no family in this group of patients in which at least one mutation would not have been found.

Two causal mutations were also found in all three patients who died shortly after birth, who clinically presented (among other things) with bilaterally enlarged kidneys with a number of small cysts and pulmonary hypoplasia. One aborted fetus with a severe phenotype, manifested by anhydramnios, enlarged kidneys with number of small cysts, dilated proximal and distal tubules as well as collecting ducts, also harbored two mutations. Therefore, this pre- and perinatal manifestation could be seen as the typical clinical picture of patients with an early manifestation of ARPKD.

The last patients with two found mutations were brothers who were included in Group B, as they do not manifest with any liver pathology. The combination of 2 missense mutations was found in their case. One (P755L) is regarded as a probably damaging mutation, the other (I222V) a mutation generally considered as pathogenic or probably pathogenic in the literature; however, found by two prediction programs (Polyphen2 and MutationTaster - see Methods) as benign/polymorphism. Since we only found this mutation in this family, we can only speculate about its benign or pathogenic significance on the ARPKD phenotype.

The majority of patients within Group B (8 of 14, 57 %) had no mutation of *PKHD1* found. There were 5 pediatric patients - 4 of them do not manifest any liver involvement, the fifth child does have hepatic pathology, but manifests extra-renal and extra-hepatic symptoms such as mild motor-mental retardation and visual impairment (suspected nystagmus in the past medical history) that do not occur in typical patients with ARPKD. We suggest that these children suffer from another cystic kidney disease other than ARPKD (*e.g.*, nephronophthisis, Joubert syndrome).

Also, no mutation was detected in two fetuses from terminated pregnancies: one with prenatal anhydramnios and autopsy findings including hypoplastic kidneys with cystically dilated collecting ducts, club foot, Potter facies, and no liver impairment (patient 974); the other with prenatal findings, such as oligohydramnios, bilaterally massively enlarged kidneys and pulmonary hypoplasia, but also with a positive family history of autosomal dominant polycystic kidney disease caused by mutation in the *PKD2* gene (which was also inherited in this fetus - patient 1629). The last patient without a *PKHD1* mutation was a fetus, the sample of which was obtained by amniocentesis, with a normal level of amniotic fluid and bilaterally enlarged hyperechogenic kidneys - nonspecific prenatal findings that can occur in several disorders (see [27, 32]).

## Conclusions

In conclusion, our study demonstrated that the detection rate of the *PKHD1* mutations in Czech children who fulfilled all three of the clinically diagnostic criteria of ARPKD is high, reaching 90 %. However, mutations of the *PKHD1* gene were also detected in some of the patients without having met all three clinical diagnostic criteria, especially in patients who died perinatally with findings such as oligo/anhydramnios, pulmonary hypoplasia, and enlarged kidneys with small cysts. The important and essential sign of ARPKD in children outside the peri- and neonatal period proved to be congenital hepatic fibrosis (or another liver pathology, such as Caroli’s syndrome), as four of five children within our study without mutation had no liver pathology.

The most frequent mutation was missense mutation T36M, accounting for 21 % of all identified mutations, and most often causing a rather severe form of ARPKD.

The next-generation sequencing method facilitates molecular diagnosis of ARPKD because of its relatively low costs and rapidity in comparison to Sanger sequencing; therefore, allowing for analyzing even those patients without all the criteria for a clinical diagnosis of ARPKD.
